# The mechanisms on evasion of anti-tumor immune responses in gastric cancer

**DOI:** 10.3389/fonc.2022.943806

**Published:** 2022-11-10

**Authors:** Junyang Wang, Tong Liu, Tao Huang, Mei Shang, Xudong Wang

**Affiliations:** Department of Gastrointestinal Nutrition and Hernia Surgery, The Second Hospital of Jilin University, Changchun, Jilin, China

**Keywords:** gastric cancer, immune evasion, tumor associated antigen, antigen presentation, tumormicroenvironment, signaling pathway

## Abstract

The immune system and the tumor have been at each other’s throats for so long that the neoplasm has learned to avoid detection and avoid being attacked, which is called immune evasion. Malignant tumors, such as gastric cancer (GC), share the ability to evade the body’s immune system as a defining feature. Immune evasion includes alterations to tumor-associated antigens (TAAs), antigen presentation mechanisms (APMs), and the tumor microenvironment (TME). While TAA and APM are simpler in nature, they both involve mutations or epigenetic regulation of genes. The TME is comprised of numerous cell types, cytokines, chemokines and extracellular matrix, any one of which might be altered to have an effect on the surrounding ecosystem. The NF-kB, MAPK, PI3K/AKT, JAK/STAT, Wnt/β-catenin, Notch, Hippo and TGF-β/Smad signaling pathways are all associated with gastric cancer tumor immune evasion. In this review, we will delineate the functions of these pathways in immune evasion.

## 1 Introduction

Gastric cancer (GC) was fifth most frequently occurring and fourth most lethal among malignant tumors worldwide as of the year 2020 (1). GC is a malignant disease characterized by a convoluted immune response, in particular to persistent inflammation. Therefore, the immune system is pivotal in cancer initiation and progression (2). As our understanding of gastric cancer’s immune-related research grows, we find that gastric cancer’s immune escape mechanism is distinct from that of other malignancies. This could point immunotherapy against GC in a novel route. In 2002, Gavin P. Dunn and Robert D. Schreiber developed the notion of tumor immunoediting, which divides the process into three stages: elimination, equilibrium and evasion (3). The preclinical tumor is killed during the elimination stage of the immune response to neoplasms. To survive the immune system’s destruction during the equilibrium phase, tumor cells may constantly mutate, for example. There is a dynamic equilibrium between the breakdown of the immune system and the growth of tumor cells. Malignant tumors, such as GC, are characterized by immune evasion, the ability of the neoplasm to elude the monitoring and attack of the immune system (4). Antigen loss or variation, a deficiency in class I Major Histocompatibility Complex (MHC I) molecules, the production of immune-suppressing cytokines, a lack of co-stimulators and other immune-suppression mechanisms are all biological processes that contribute to immune evasion (5). We classify them as follows: loss or alterations in tumor-associated antigen (TAA), damage to the antigen presentation mechanism (APM), and immunosuppression by the tumor microenvironment (TME). The immune system plays a crucial role in tumor development, hence researchers are looking into using immunologic techniques to increase the longevity of GC patients. Despite the fact that tumor immunotherapy (especially immune checkpoint inhibitor) has had a lot of achievements, many patients do not respond to treatment and many cases become resistant to treatment. For this reason, it is essential to always be on the lookout for cutting-edge therapies. Multiple signaling pathways have emerged as possible contributors to immune evasion. Inhibitors of signaling pathways may therefore form part of future immunotherapies. Consequently, the signaling pathway is highlighted as a crucial component of GC’s immune evasion strategy in this paper.

## 2 The mechanism of immune evasion

In order to escape immune monitoring and eradication, tumor cells modify their TAA and APM in a number of ways, then entering the final evasion stage (5). Tumor cells that have evaded the immune system are able to survive and have an effect on the tumor microenvironment (TME) through a number of signaling pathways, dampening the anti-tumor immune response (6).

### 2.1 Escaping surveillance by invalid TAA

To a certain extent, TAA can be divided into three categories (7): first, TAA presented on the surface of tumor cells by major histocompatibility complex (MHC) molecules or antigen presenting cells (APCs) and recognized by autoantibody or heteroantibody; second, target molecules or ligands recognized by natural killer (NK) cell receptors; and third, TAA presented on the surface of tumor cells by MHC molecules or APCs and recognized by autoantibody or heteroantibody (APCs). Correspondingly, TAA’s modulation can also be divided into three groups: 1) the up-regulation of immunosuppressive antigen; 2) the loss of original recognized antigen; 3) the generation of unrecognizable antigens (4). Antigen expression is known to be influenced by epigenetic regulation, as well as mutations in genes (8). A genetic mutation is any alteration to the gene’s base pair sequence or makeup. Epigenetic regulation is the heritable alteration of gene expression that does not involve a change in nucleotide sequence and includes DNA methylation, histone modification, and regulation by non-coding RNA (ncRNA). Gene silencing occurs when DNA is methylated at the C-terminus of 5’-CpG-3’ by DNA methyltransferases (DNMTs) to produce 5-methylcytosine (5-mC) (9). Enzymes collaborate to modify histones in various ways, including methylation, acetylation, phosphorylation, ubiquitination and ADP ribosylation (10). Complexity of the regulation mechanisms of ncRNAs in immune evasion will be detailed in the later section.

Carcinoembryonic antigen (CEA), HER2, carbohydrate antigen 19-9 (CA19-9) and CA72-4 are some of the most common GC autoantigens. They are highly expressed, but their immunological impact is quite modest (11). The main function executor is a member of the CEA family called CEA-related cell adhesion molecule-1 (CEACAM1). Evidence suggests that CEACAM1 inhibits NKG2D ligand (NKG2DL) expression in tumor cells (11). To make tumor cells more susceptible to NK cell-mediated cytotoxicity, Chen et al. (11) found that silencing CEACAM1 in mice and human tumor cells increases surface NKG2DL expression. Since HER2 belongs to a member of the EGFR family, it can also activate the downstream PI3K/AKT and ERK pathways, which in turn control cell proliferation, invasion and migration (12). Previous studies have shown that CD8+ cytotoxic T lymphocytes (CTLs) can recognize HER2, which aids in the immune system’s fight against tumors (12). A recent study by Wu et al. (13) demonstrates, however, that HER2 protects cancer cells from STING-mediated innate antitumor immunity by activating AKT1, suggesting that HER2 recruits AKT1 to lower STING signal, hence restricting anti-virus defense and anti-tumor immunity. Nevertheless, the mechanism by which CA19-9 and CA72-4 play a role in immune escape remains unexplored at this time and warrants further investigation.

The main activating receptor expressed by NK cells, NK group 2D (NKG2D), binds to ligands such as MHC class I peptide related sequence A (MICA), MICB and six UL16 binding proteins (ULBPs) (14). The primary ligand on the surface of tumors is a variant of the traditional MHC protein called MIC A/B. Both β2-microglobulin (β2m) and antigen are inaccessible to MIC A/B (14). Gene promoter hypermethylation, histone deacetylation and protein shedding contribute to reduced MIC A/B expression on tumor surfaces (15, 16), hence dampening NK cell-mediated innate immunity.

Immune checkpoints (ICPs) and co-inhibitory molecules (CIMs) consist of ligands and their respective receptors. Particularly, these ICPs are focusing on the PD-1/PD-L1 field. PD-L1 on the tumor surface interacts to PD-1 on the T cell surface, leading to T cell depletion (17). Studies show that PD-L1 is highly expressed on the surface of tumors, and its induction process is complex. Helper T (Th) cells, cytotoxic T lymphocytes (CTLs) and natural killer (NK) cells that have been activated can all generate interferon (IFN), which can then activate the JAK/STAT pathway and lead to PD-L1 expression. In the meantime, IL-10 can boost PD-L1 expression (12, 18). Moreover, the C > G variant of the rs4143815 SNP in the 3’-UTR of the PD-L1 gene increases PD-L1 expression and may increase cancer risk (19). Tumor-associated macrophage (TAM) production of tumor necrosis factor alpha (TNF-α) and interleukin-6 (IL-6) positively regulated PD-L1 (20). High expression of MHC II in GC cells can be partially explained by the lack of traditional co-stimulatory proteins CD80 and CD86 in the tumor, which limit MHC class II recognition (21). In addition, researchers discovered a decreased expression of co-stimulatory molecules such as 4-1BBL (tumor necrosis factor receptor superfamily member 9 ligands), B7-1 and CD40 on the tumor surface (22–24). ICPs inhibitors paired with co-stimulatory molecular agonists may be a viable way for tumor treatment, according to these studies. CD47, also called integrin-related proteins, is a cell surface glycoprotein of 50-kDa that inhibits APM induced by macrophages through the “don’t eat me” signaling CD47/SIRP pathway (25). Macrophage-mediated innate immune and APM inactivation is aided by the CD47/SIRP (signal-regulatory protein) axis, which inhibits phagocytosis by downregulating integrin signal activation from the interior of macrophages (26). The MYC mutation causes CD47 upregulation and contributes to PD-L1 overexpression in a similar fashion (27). The work by Yoshida K et al. (25) found that GC that express the surface marker CD47 proliferated strongly in both vitro and *in vivo*.

Factor-associated suicide (Fas) is a member of the tumor necrosis factor (TNF) family of type II transmembrane proteins. It is also known as CD95 or apoptosis antigen-1 (Apo-1). This protein has the ability to connect with its ligand, set off the apoptosis cascade, and maintain a pro-apoptotic environment (FasL) (28). Activated T and NK cells are the most common sources of FasL. Wang et al. found that low levels of Fas expression on the surface of GC cells were associated with a poor prognosis *in vitro* studies (29). The rs2234767 G> A polymorphism in the Fas promoter region may be associated with susceptibility to GC (30), which may be associated with SNP-induced down-regulation of Fas. Similarly, epigenetic changes can affect Fas expression. Fas expression was downregulated due to hypermethylation of its promoter region (31).

### 2.2 Escaping surveillance by damaged APM

Antigen presentation involves antigen processing and degradation by APCs such macrophages and dendritic cells (DCs), followed by presentation of the antigen peptide/major histocompatibility complex (MHC) complex to T lymphocytes (32). APCs deliver antigen polypeptides by joining them with processed MHC class II for specific recognition by CD4+ T lymphocytes (33). CD8+ T lymphocytes, and in particular CTLs, are capable of direct MHC class I detection (34).

The major histocompatibility complex (MHC) family is a group of membrane proteins responsible for presenting antigens on cell surfaces, where they can be recognized by T lymphocytes, which then kill the cell (35). The human MHC locus, also known as the human leukocyte antigen locus, is found on chromosome 6 and contains around 200 genes (HLA) (35). Many malignant tumors include aberrant expression of class I and class II molecules, the primary types responsible for presenting antigens to T lymphocytes (36). HLA class I molecules consist of the heavy chains (HLA-A, -B, -C, -E, -F and -G) and the β2m (37). Changes in epigenetic regulation and a mutation in the HLA gene, called β2m, are primarily responsible for the dramatic reduction in class I HLA expression (38–40). Down-regulation of HLA class I is caused by hypermethylation of the promoters of the HLA-A, -B and -C genes, which is a hallmark of GC (39, 40). An example is the finding by Ye et al. that promoter methylation is linked to reduced HLA-A expression in BGC-823 cells (40). Recent research has indicated that HLA-G is overexpressed, leading researchers to hypothesize that non-canonical HLA class I may have a deleterious effect in GC due to unidentified antigen (41). EZH2 (enhancer of zeste homolog 2) is a major component of Polycomb inhibitor complex 2, which catalyzes histone H3 lysine 27 trimethylation (H3K27me3) (42). Activation of EZH2 in tumors results in H3K27 methylation, which in turn silences key immune genes including HLA class I (42). The HLA class II trans-activator promoter is associated with epigenetic control of HLA class II (CIITA) (43). Decoy receptor 3 for interferon beta and tumor necrosis factor (TNF) inhibits HLA class II (mostly HLA-DR gene) expression by hypermethylation and histone deacetylation of CIITA-Promoter IV (CIITA-PIV), which is activated by STAT1 and requires histone deacetylases (HDACs) (DCR3) (41). HLA class II antigen presentation stimulates Th cells activation without co-stimulatory molecule, and its upregulation has been described in some forms of GC (41). Antigen presentation can be improved by binding TAA to HLA, but this cannot happen without TAP (transporter associated with antigen processing) and tapasin (TAP binding protein) (44). A decrease in histone H3 acetylation and TAP1 expression is caused by the decreased binding of histone acetyltransferases (HATs) to gene promoters, which in turn decreases the accessibility/transcription of the RNA polymerase II complex (27). When EZH2 is turned on in a tumor, TAP1 and TAP2 are also suppressed (42). The results of these studies provide evidence that inhibiting key enzymes that regulate epigenetics may be an effective treatment for GC.

**Figure 1:**
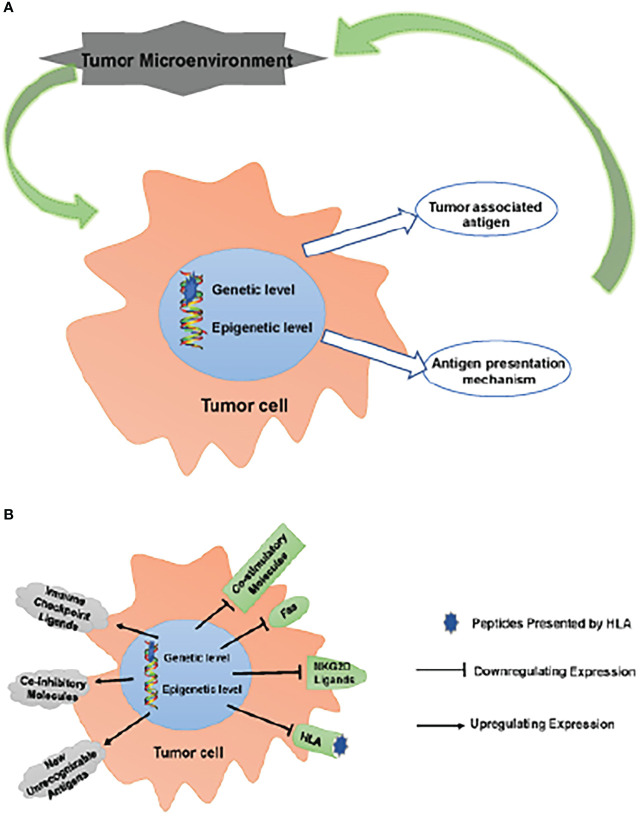
Escaping immune surveillance by changing TAA and APM. **(A)** Tumors have altered self-antigen expression through genetic mutations or epigenetic regulation, resulting in tumor cells escaping immune surveillance and clearance. Tumor cells that have escaped clearance survive to form an immunosuppressive microenvironment together with surrounding cells/cytokines, which further promotes tumor survival. **(B)** These changes up-regulate inhibitory molecules, such as ICPs (PD-1, CTLA-4, LAG-3 and TIM-3) ligands, CD44 and CD47; meanwhile, new unrecognizable antigens appear. On the other hand, the expression of co-stimulatory molecules, CD40, Fas, NKG2D ligands (MICA/B, ULBPs) and HLA is down-regulated. These changes lead to the obstacle of TAA and APM, which makes tumor cells escape immune surveillance.

APM impairment impacts the TME as a result of a combination of fewer invading APCs and their malfunction, which means that tumor cells are able to evade immune monitoring and clearance due to the combined effects of inefficient TAA and defective APM (45). Those tumor cells that are able to avoid being eliminated by the immune system join with other local cells and cytokines to create an immunosuppressive microenvironment that aids in the growth and survival of the tumor. (**Figure 1**)

### 2.3 Signaling pathways involved in immune evasion

The term tumor microenvironment (TME) encompasses everything from other cells to their secretions to the metabolites they create. The signal route of tumor infiltrating lymphocytes (TILs) can be further regulated and inhibited by inflammatory substances secreted by tumor cells (**Table 1**). Tumor cells are able to evade immune surveillance when the early microenvironment regulates many signaling pathways, resulting in a diminished anti-tumor immune response and, ultimately, immune suppression (92).

**Table 1:** The main component and function of TME.

component	function	ref
MSCs	recruiting CAFs, M2 macrophage and IL-6; maintaining an inflammatory milieu	(46–49)
MDSCs	limiting DC maturation and inducing Treg generation due to its accumulation; suppressing T cell and NK cell activation	(21, 50–53)
M1 macrophage	secreting pro-inflammatory factors through IFN-γ and LPS activation in the early stage of inflammation	(46, 47, 49, 52, 54, 55)
M2 macrophage	inhibiting inflammatory reaction through IL-4, IL-13 activation	(46, 47, 49, 52, 56, 57)
CAFs	secreting IL-6, IL-8, VEGF, CXCL9 and TGF-β to inhibit T cell function	(49, 51, 58–60)
Th1 cell	promoting inflammatory response viasecreting IL-2, IFN-γand TNF-αin the early stage of inflammation	(61–64)
Th2 cell	inhibiting Th1 cell proliferation and inflammatory reaction	(53, 61, 63, 65)
Th17 cell	promoting inflammatory response *via* secreting IL-17, IL-22;participating in maintaining Treg/Th17 cells balance	(66–69)
Treg	regulating the inflammatory response to be chronic; secreting inhibitory cytokines to inhibit immune response; inhibiting the proliferation and activation of Teff cells and Th1 cell	(65, 66, 68, 70, 71)
DCs	antigen presentation	(72–77)
Fas/FasL	inducing apoptosis of activated Teffcells to escape immunerecognition andelimination	(28–30, 51, 62, 78)
PD-1/PD-L1	exhausting Teff cells	(18, 66, 79–82)
IL-6	mainly secreted byTh2 cells,CAFs and tumor cells;activating STAT3 andNF-kBpathwaytochange its phenotype to variants	(20, 68, 83–85)
IL-8	mainly secreted by MSCs andCAFs;stimulatingPD-L1expression viaSTAT3andmTORpathway	(46, 86)
IL-10	mainly secreted byTh2cells andTreg;inhibiting the proliferation and activation ofTeff;increasing infiltration ofTAMs, MDSCs, Th2 cells andTreg;stimulatingPD-L1expression	(18, 65, 87, 88)
IL-17	intensifying inflammatoryresponse *via* activatingNF-kB and MAPK pathway; promoting the activation ofT cells and the secretion of IL-6,IL-8 and GM-CSF	(66, 67, 83, 89)
IL-33	activating NF-kB and MAPK pathway; promoting inflammation and the secretion of GM-CSF	(53, 90)
CXCL12/CXCR4	promotingEMTandpreventtumor cell death;promotingMDSCs accumulation;regulating PI3K/mTORpathway	(51, 91)

#### 2.3.1 NF-kB signaling pathway

The nuclear factor kappa B (NF-kB) pathway plays a crucial role in modifying the immune response to infection, especially in chronic inflammation. This pathway is composed of two main subgroups: 1) NF-kB1/NF-kB2 (p50/p52); 2) Rel A (p65), Rel B and c-Rel (93). Toll-like receptors (TLR), EGF, PI3K, IL-1 and TNF can all activate the NF-kB signaling pathway (93). Interleukin-6 (IL-6), tumor necrosis factor (TNF), T helper 2 (Th2) cells, regulatory T cells (Treg), type 2 (N2) neutrophils, myeloid-derived suppressor cells (MDSCs) and mesenchymal stem cells (MSCs) are all up-regulated when the NF-kB p65 or c-Rel pathway is active (46, 47, 50, 83, 94). Recently, O’Reilly found that NF-kB1 has anticancer qualities since activating the STAT1 pathway increased GC growth in NF-kB1^-/-^ mice by decreasing TAP gene expression and inhibiting innate immunity (79). Furthermore, deleting NF-kB1 also increased the expression of CTLA-4 and PD-1 in lymphocytes and the expression of programmed death ligand-1 (PD-L1) in myeloid and gastric epithelial cells (79). Similarly, HLA class II was up-regulated in GC epithelial cells from NF-kB1^-/-^ mice (79).

Tumor-induced MSCs interact with neighboring cells in the tumor microenvironment (TME) to promote tumor progression (48). The exosomes secreted by GC cells modulate the immunomodulatory activity of MSCs *via* the NF-kB signaling pathway, thereby boosting MSCs’ capacity to activate immune cells, sustaining an inflammatory milieu and promoting tumor growth (49). In addition to regulating angiogenesis and morphogenesis, mesenchymal stromal cells (MSCs) have been demonstrated to recruit cancer-associated fibroblasts (CAFs), IL-6 and M2macrophage, all of which have been linked to cancer progression (46, 49, 83). Examples include MSC-derived M2 macrophages, which express vascular endothelial growth factor (VEGF) in an NF-kB p65-dependent manner (93), MSC-derived IL-6 activating neutrophils, which in turn increases angiogenesis and tumor spread (84), and tumor-derived factor being able to polarize neutrophils to the N2 phenotype. N2-polarized neutrophils support metastasis and inhibit the immune system (95). Different kinds of circulating neutrophils include high-density neutrophils (HDN) and low-density neutrophils (LDN) (96). According to research by Sagiv JY et al. (96), LDN is associated with cancer and is induced by HDN through activation of transforming growth factor (TGF), which promotes tumor progression. The TME of GC also inhibits apoptosis in neutrophils and promotes the production of inflammatory molecules like IL-1 and TNF-α, dampening the immune response (97). Furthermore, TNF-α also increases CD47 expression *via* the NF-kB signaling pathway at the transcriptional level (98). Since MSCs have the ability to regenerate, they are a promising tool in the fight against cancer (51). CAFs are derived from bone marrow-derived stem cells, pericytes and normal gastric fibroblasts stimulated by TGF–β. A possible contributor to the development and spread of cancer is the accumulation of CAFs in GC tissue (51). Also, CXC motif chemokine ligands 1/2 (CXCL1/2) can be induced by TNF in an NF-kB-dependent manner in stromal cells and endothelial cells (51). On the one hand, IL-8 produced by CAFs increases cisplatin resistance in GC *via* activating NF-kB p65 and binding CXCR1/2 (46, 53). On the other one hand, IL-17 can heighten the inflammatory response by stimulating NF-kB p65 and MAPK, which in turn increases IL-8 secretion (83). These cytokines cause the production of S100A8/9, a calcium-binding protein with a small molecular weight. High levels of S100A8/9 are found in inflammatory conditions; these conditions are related with a decrease in DCs and an increase in MDSCs (90). Also, S100A8 can stimulate the expression of PD-L1 and the polarization of TAMs from M1 to M2 (90). In contrast to M1 macrophages, which suppress antitumor immunity, M2 macrophages have been shown to have an Immunosuppressive effect. Furthermore, Probst et al. (99) discovered that immature DCs can enhance CD8+ T cell tolerance through the PD-1 and CTLA-4 molecules.

MDSCs, immature cells derived from bone marrow that can grow into dendritic cells, macrophages and granulocytes once enlarged, recruited and activated. In turn, an increased number of MDSCs can suppress DC maturation, lead to the production of Tregs, and ultimately dampen the immune response (51). Additionally, the presence of FasL on activated T lymphocytes mediates the activation of the Fas signal in tumor cells (51). It induces prostaglandin E2 (PGE2) secretion from tumor cells, which in turn increases tumor cells’ potential to entice MDSCs (51). By regulating arginine and tryptophan metabolism with the help of arginase, inducible nitric oxide synthase, and indoleamine-2, 3-dioxygenase 1 (IDO1), MDSCs are also able to inhibit the activation and proliferation of T cells and NK cells (51). IL-33, a member of the IL-1 family, was found to increase the immunosuppressive capacity of MDSCs by stimulating the up-regulation of arginase-1 and blocking the death of MDSCs by enlisting MSCs (53). On the other hand, IL-33 expression was up-regulated in MDSCs following NF-kB activation. While IL-12 expression was down-regulated, M2 macrophage and Th2 cell polarization was enhanced (53). A novel and effective treatment may be IL-33 therapy in combination with NF-kB inhibitors.CD8+ CTLs, an HLA class I co-stimulatory molecule, are a major effector cell for eliminating tumor cells (90). Unlike the theoretical effect, IFN-γ generated by CTLs can also cause MDSCs to clump together, inhibiting the activation and proliferation of T and NK cells (90). IFN-γ can also up-regulate the expression of IDO1 from the transcriptional level (99). In addition, an active NF-kB pathway can increase CD36 transcription and fatty acid (FA) absorption activity, decreasing DC numbers, by directly modifying the s468 and t470 sites of CD36 (100, 101). CD36 prevents Treg cells from committing apoptosis and boosts Treg cell activity in specific settings (102). In addition, a rise in Treg cells occurs when NF-kB c-Rel is activated (94). Interestingly, vasoactive intestinal peptide (VIP) generated by GC cells increases responsiveness of Th2 cells, lowers proliferation of Th1 cells, interferes with the formation of B cells and suppresses the activity of NK cells by down-regulating NF-kB p65 (61). Th2 cells, on the other hand, often boost and repress the activation of effort T cells, which is counter to the antitumor effect of Th1 cells. There is a direct correlation between NF-kB pathway activation and the suppression of Fas expression on tumor surfaces (62), which in turn leads to a decrease in tumor cell death and an endless proliferation of tumor cells. Because of its significance in the immune response, inhibiting the NF-kB pathway may be helpful.

#### 2.3.2 MAPK cascade

The mitogen-activated protein kinase (MAPK) cascade controls a wide variety of physiological and pathological processes, including cell proliferation, differentiation, stress and inflammation (103). After cells were stimulated by receptor protein tyrosine kinases (RPTKs) like growth factors (GFs), chemokines, or other stimuli, MAPK was activated by increased phosphorylation (66, 103). The MAPK pathway is separated into three branches: p38 MAPK, ERK and JNK. Extracellular regulated protein kinase (ERK) is largely activated by EGF (104). c-Jun N-terminal kinase (JNK) and p38 MAPK signaling are triggered by numerous stress stimuli, including ROS and inflammatory cytokines (such as TNF–α, IL-1β and IL-18), causing inflammation and apoptosis (70, 104, 105).

Both immune-suppressing cells (MDSCs and Treg) and immune-promoting cells (Th17 cells) can proliferate in response to GFs, chemokines and Ras mutations, which activate the ERK cascade (66). The pro-inflammatory cytokines IL-17 and IL-22 are produced mostly by Th17 cells, which evolved from Th0 cells (67). Differentiation of Th17 cells is encouraged by TGF-β, IL-1, IL-6, IL-21 and IL-23 (67), but is inhibited by IFN-γ, IL-2 and IL-4. Treg and Th17, both CD4+ T cells, make up a balance system, and their breakdown is intimately linked to inflammatory immunosuppression in cancer (66). TGF-β is a bidirectional cytokine that, in the late stages of cancer, induces Treg and Th17 cell development from naive T cells to protect tissues against an overactive immune response (68). This functional change of TGF-β is interesting to investigate because it may offer a novel explanation for the degradation of the microenvironment. Inducing Treg cell differentiation and suppressing Th17 cell proliferation are two ways in which GC generated MSCs have recently been shown to reduce antitumor immune responses (69). C-X-C motif chemokine ligand 8 (CXCL8, IL-8) increases the malignant phenotype of GC cells, yet IL-17, which is produced by GC, can stimulate the transition of normal fibroblasts into CAFs by stimulating NF-kB signaling (106). Whether IL-17 produced by GC cells or Th17 cells serves the same purpose is currently unknown. Therefore, the role of Th17 cells in the immunological response to GC is yet to be investigated. Surprisingly, the route also increases the expression of ICPs (such as PD-1, LAG3 and CLTA4) on T cell surfaces, depleting effector T cells (Teff). PD-L1 and PD-L2 can be up-regulated at the transcriptional level by oncogenic mutations of Ras or EGFR (66). Besides, inflammatory chemicals, especially IFN-γ, often upregulate PD-L1 expression, a phenomenon known as adaptive immune resistance (81). Even though IFN-γ exerts anti-neoplastic effects in the early stages of tumor development, it has been shown that chronic IFN-γ activation of tumor cells suppresses T cells and leads to the accumulation of MDSCs (107). If we can better understand how IFN produces MDSCs, we may be able to employ interferon more effectively to treat tumors. Apoptosis in CD3+ T cells, the most abundant group of T cells, is an indication of T cell dysfunction and weakening. Apoptosis of CD3+ T cells, the main subgroups of T cells, can be induced *via* the PD-1/PD-L1 axis, when KRAS mutations increase PD-L1 (81). When the MAPK pathway is in charge of IL-10’s activity, it can decrease CD8+ T cells and promote Treg-mediated immunological tolerance to cancer (70, 87). Tumor immunosuppressive cells including M2 macrophages, MDSCs and Treg can multiply while effector CD4+ and CD8+ T lymphocytes are inhibited from doing so by IL-10 and TGF-β (66). Tumor-derived cytokines such as PGE2, IL-10, IL-1, TGF-β and VEGF may induce the differentiation of immature myeloid cells (CD33+ cells) into MDSCs (87, 108). In addition, IL-1, IL-6 and IL-17 are believed to increase production of CXCL12, which can recruit MDSCs (89, 91). CXCL12 is produced in the stomach mucosa in response to inflammation, and this helps CXCR4+ MSCs and CAFs migrate. The elevated levels of CXCL12 promote EMT and inhibit tumor cell death by upregulating CXCR4 and CXCR7 in a positive feedback loop (59). Additionally, PGE2 can promote MDSC recruitment by stabilizing CXCR12 and activating chemokines including CXCL12 and CXCR4 (51). These results suggest that a combination of DC immunization and measures to decrease MDSCs accumulation is an effective way to treat tumors.

A MAPK cascade is useful for dampening the anti-tumor immune response (65). A decrease in the expression of DCs-related molecules such CD40, CD80, CD86 and IL-12 was observed, while an increase in IL-10 secretion was observed (72, 85). IL-12 is a critical cytokine for T cell activation and DC maturation and survival (73). It has been established that TGF-β, IL-6, IL-10 and VEGF all work together to produce tolerant DCs, which in turn promotes the growth of Th2 cells and Tregs (65). For instance, Marigo et al. demonstrated that IL-10 can transform naive T cells into Treg *in vivo* and *in vitro*, facilitating immune evasion (66, 71). Another need for CD8+ T cell death is active p38 MAPK (109). Expression of TNF-α, IL-6 granulocyte macrophage colony-stimulating factor (GM-CSF) are all controlled by p38 MAPK (110). Previous research has shown that the cytokines GM-CSF and IL-6 can rapidly produce MDSCs from bone marrow progenitor cells in both mice and humans (111). The therapeutic efficacy of a tumor vaccination can be diminished by the presence of tumor-derived GM-CSF, which suppresses apoptosis in MDSCs that are linked with tumors. The up-regulation of arginase-1 that is induced by IL-33 is another way in which this autocrine GM-CSF signal of MDSCs is amplified (53). Moreover, oxidative stress may activate the p38 MAPK pathway to down-regulate NKG2DL, including MICA/B and ULBP1-4 (11). In addition to its role in tumor evasion, the JNK pathway is essential for its maintenance. For instance, aberrant tumor glycolysis promotes JNK pathway expression, which in turn promotes PD-L1 expression (112, 113). Besides, IL-18 promotes tumor cell adhesion, migration, invasion and angiogenesis *via* the JNK pathway, which leads to an increase in thrombospondin 1 (TSP-1) (105). Besides, Kim and his team demonstrated that down-regulating Fas expression on the tumor surface *in vitro* by activating the JNK/p38 MAPK signaling pathway (78). Hence, MAPK inhibitors could be utilized to treat patients by decreasing the number of MDSCs and immature DCs that have accumulated in the body.

#### 2.3.3 PI3K/AKT signaling pathway

Protein kinase B (AKT) is triggered in response to phosphatidylinositol 3-kinase (PI3K) activation, and once in the nucleus, it affects cell proliferation, invasion, metabolic reprogramming, migration, autophagy, senescence and carcinogenesis. Besides, the non-classical NF-kB signaling pathway can also be activated by AKT (82). When the phosphatase and tension homolog deleted on chromosome 10 (PTEN) gene is lost or mutated, a negative regulator of AKT is turned off, leading to PD-L1 overexpression in cancer (82, 114). T cell proliferation and effector function are inhibited by PD-1 and PD-L1 or PD-L2 interaction, which also induces apoptosis and encourages the conversion of CD4+ T cells into Foxp3+ Treg cells (88). In contrast, PD-1 up-regulates FasL and increases IL-10 production, which further suppresses the immune response (88). For example, TGF-β signaling enhances Treg cell activity by upregulating Foxp3 expression under the chronic inflammation (115). Inducer of the epithelial-mesenchymal transition (EMT) that can upregulate pro-inflammatory cytokines including IL-1, IL-6 and IL-8 to improve immune cell chemotaxis and migration is SNAIL (115), whose gene transcription is promoted by AKT activating NF-kB (115). By the way, IL-8 from MSCs increases PD-L1 expression in GC cells *via* the c-MYC signaling, which is regulated by the STAT3 and mTOR signaling pathways (86). In addition, AKT-supported immune evasion enhances the activity of immunosuppressive Treg cells by making them more resistant to CD8+ T cell-mediated death (115). AKT activation of NF-kB increases the migration of Th17 cells to TME, which are primarily regulated by C-C motif chemokines ligands 20 (CCL20) (91). C-C motif chemokine 20 (CCL20) has been found to have a crucial role in cancer as a mediator by interacting with C-C motif chemokine receptor 6 (CCR6) (116). Moreover, the presence of both CXCL12 and CXCR4 in gastric adenocarcinoma promotes GC invasion by up-regulating the PI3K/mTOR pathway and the MET process (91). Finally, through modulating Treg differentiation and PD-1/PD-L1 expression, the PI3K/AKT signaling pathway facilitates immune evasion. T cells, B cells and NK cells can all have their activation and proliferation suppressed by CD4+ Treg and CD4+ Treg can also attract MDSCs in the tumor stroma (117). For instance, stopping CD8+ T lymphocytes from being recruited to malignancies can be achieved by stimulating the PI3K/AKT/mTOR signal in M2 macrophage (118). High frequencies of Treg cells and low numbers of Teff were found to be characteristic of GC, as revealed by Kumagai’s research (119). While glucose deprivation is lethal to CD8+ T cells and conventional CD4+ T cells, RhoA Y2-mutation increases the PI3K/AKT/mTOR signaling pathway, increasing the quantity of free fatty acids (FFA) in the TME, allowing Treg cells to survive and operate under FFA metabolism, which demonstrates that RhoAY42-mutant GC is not a promising candidate for PD-1 blocking monotherapy (119). Still, Targeted PI3K and PD-1 inhibitor combo therapy still outperforms PD-1 inhibitor therapy alone.

#### 2.3.4 JAK/STAT signaling pathway

Janus kinase (JAK) rapidly recruits and catalyzes the tyrosine phosphorylation of signal transducers and tyrosine activators (STAT) situated on the receptor after receiving a signal from upstream receptor molecules. As soon as these receptors are activated, STAT proteins bind to them *via* the SH2 domain and translocate to the nucleus, where they control the transcription of specific genes (120). Interestingly, if JAK stimulates SHP-2, it may enter the MAPK cascade, and if PI3K is active, the PI3K/AKT pathway is initiated (120). Conversely, the p38 MAPK cascade can activate downstream STAT1. In addition to promoting carcinogenesis, STAT3 activation can block STAT1-mediated tumor suppression (121). In spite of this, STAT1 is typically regarded as a tumor suppressor. Intriguingly, Gabrilovich discovered that inducible nitric oxide synthase (iNOS) and arginase-1 overexpression in TAMs suppressed T cells *via* activation of STAT1 (122). Similar to what we see with PD-L1 expression *in vitro*, O’Reilly discovered that activating the STAT1 pathway may also enhance GC formation in NF-kB1^-/-^mice (79).

The JAK/STAT cascade was first discovered in the IFN-α, IFN-γ and IL-6-mediated signaling pathways (123). What’s more, IL-8, IL-17, IL-22, TGF-β, GM-CSF and EGF all stimulate the JAK/STAT pathway as well (124, 125). Activation of STAT3 results in increased expression of the genes encoding for Th17, M2 macrophage, MDSCs, Th2, Treg, PD-L1 and IDO 1 (86, 120, 121, 126). The expression of IL-6 is triggered by the aromatic hydrocarbon receptor (AHR) being activated by indoleamine IDO1 (82). At the same time, IDO1 activity can keep its expression continuing *via* the autocrine Kyn/AhR/IL-6/STAT3 signal loop (126). STAT3 and NF-kB were also activated by the Ras/Raf/MEK pathway, which led to the expression of IL-1, IL-6, IL-10, TNF and VEGF (127). This is because cytokines belonging to the IL-10 family block APCs, which in turn impedes CLT function and promotes Treg formation (127). Foxp3 expression by Tregs is dependent on STAT5 activation, which in turn is required for the production of GM-CSF-stimulated T cells (124, 127). Studies showed that immunosuppressive cytokines and cells accumulated in the TME due to the JAK/STAT cascade’s primary role in this process. Given these results, we believe that STAT inhibitor is a potential drug and may one day be used to treat GC by enhancing positive TME.

#### 2.3.5 Wnt signaling pathway

The Wnt signaling pathway is essential for the maintenance of pluripotency in stem cells, the regulation of embryogenesis, homeostasis, regeneration, the formation of malignant tumors and more (128). The primary objective of immunotherapy is to induce an immunocompetent response within the tumor microenvironment in order to improve recognition of the tumor, destruction of tumor cells, and responsiveness to treatment. Recent years have revealed a number of Wnt signaling pathways to be involved in immune evasion and immunological control of cancer (129).

The Wnt/β-catenin pathway relies heavily on β-catenin as a signaling molecule. Abnormal activation of Wnt/β-catenin was linked to an increase in Th2 cells, Tregs, tolerant DCs and PD-L1 (63, 74). Meanwhile, CD8+ T cell infiltration and IFN-γ release were also both suppressed by the abnormal activation of the Wnt pathway (63, 130). Furthermore, previous studies demonstrated that CD4+CD25+β-catenin+Treg cells were more robust and competitive than control Treg cells *in vivo* (131). The β-catenin/TCF4 signaling pathway induces the production of immature DCs and Treg cells phenotypes through metabolizing vitamin A to produce retinoic acid (132). Similarly, the immunosuppressive effect of over-activated Wnt/β-catenin pathway on DCs and CTLs in human melanoma has been shown by Yaguchi’s team (133). Ample evidence indicates that abnormally activated Wnt/β-catenin pathway up-regulated the expression of PD-1, thereby inhibiting the infiltration of T cells in the immune environment (134). The loss of Teff cell infiltration was linked to improperly active Wnt signal transduction, and this relationship was frequently accompanied by gene mutation and abnormal methylation, according to a large-scale genomic analysis of tumor samples (135). A recent bioinformatics analysis found that the abnormal activation of tumor cells intrinsic Wnt/β-catenin signaling is critically important in non-T cells infiltration tumors (136), which is of great significance for the treatment of immune desert tumors. For example, Wang et al. demonstrated that the β-catenin/TCF inhibitor iCRT14 significantly suppressed tumor growth *via* effectively enhancing the infiltration of T and NK cellsin anexperimental model of T cell deficiency (137). However, it is more important to determine the sequence of Wnt pathway’s abnormal activation and T cell infiltration in immune desert tumors. Besides, its abnormal activation also prevented CD4+ T cells from developing into Th1 and Th17 cells (63). In addition, the Wnt pathway regulates multiple immune cell functions, including those of MDSCs and NK cells, that plays a crucial role in cancer immune editing (138). Studies have demonstrated that the Wnt pathway is primarily responsible for cell cycle progression and the production of aberrant proteins that might cause cancer in cells. The Wnt pathway’s mechanism has recently been refined thanks to studies in the field of immunology. There is a stronger infiltration of Treg cells, immature DCs and lower Teff cells after activation of the Wnt pathway (139, 140).

#### 2.3.6 TGF-β signaling pathway

Differentiation, apoptosis, migration and other cellular activities are all controlled by the TGF-β signaling pathway, which is produced in the TME and secreted by tumor cells and stromal cells (141). Therefore, this pathway and its interruption play an important role in tumor suppression or promotion (142). The function of TGF-β mainly involves two pathways, namely the canonical pathway (Smad-dependent) and the non-canonical pathway (Smad-independent). When it comes to canonical pathway, the drosophila mothers against decapentaplegic protein (Smad) are essential. Activated R-Smads join with Smad4 to create a heteromeric Smad complex, then entering cell nuclear. It regulates gene expression by binding transcription factors and transcription co-regulators (143). Restoring tumor immunity *in vivo* may be as simple as blocking the TGF-β signaling pathway in CD8+ T cells, as discovered by Thomas et al. (144). TGF-β/Smad pathway also aided in the infiltration of Treg cells (145). These findings support the hypothesis that the TGF-β/Smad signaling pathway, by suppressing immune responses, promotes cancer. In addition, the tumor cells activate TGF-β signaling, which alters a major component of the TME known as CAFs, which in turn alters the extracellular matrix (ECM) in a way that rejects immune cells and may affect immunotherapy responses (146). Besides, TGF-β also operates on both ends of the NKG2D axis, and studies have demonstrated that it substantially inhibits NKG2D-mediated tumor killing (147).

#### 2.3.7 Other signaling pathways

**Figure 2:**
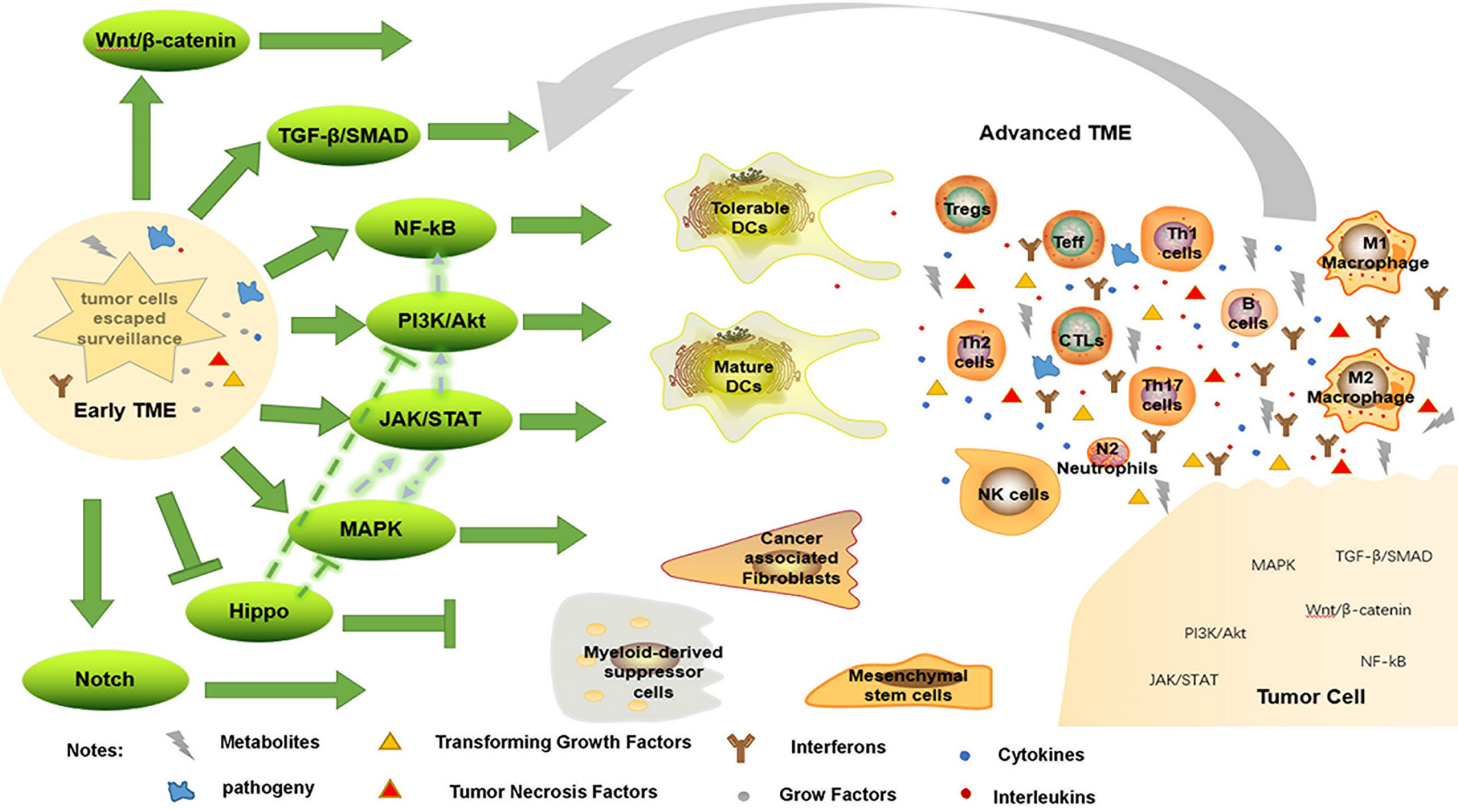
Immune evasion by changing TME. When tumor cells escape immune surveillance, they form early TME, enhancing immunosuppressive environment, weakening anti-tumor response, and further promote tumorigenesis through a variety of signaling pathways. Conversely, this advanced TME promotes tumor progression through these pathways.

Notch and Hippo pathways are also engaged in immune evasion; high levels of Notch receptor expression correspond with the presence of immature DCs, M2 macrophages, N2 neutrophils and CD4+ T cells in GC tissue (75). Similarly, high expression of Notch3 is associated with low infiltration of activated CD8+ T cells in TME (140). To decrease tumor growth, Hippo signaling has been studied extensively. Depletion of CD8+ CTLs and elevation of FoxP3+ Treg have both been linked to interference with the Hippo pathway (148). However, Hippo pathway inactivation can activate EGFR, which then activates the PI3K/mTOR and Ras/Raf pathways (149). As a result, one therapeutic strategy involves focusing on molecules that set off the Hippo pathway. In short, these intricate pathways work together to keep the surrounding tissue hospitable to tumor growth (**Figure 2**).

## 3 Impact of ncRNAs on immune evasion

As we will see, ncRNAs are an integral part of the epigenetic regulatory process, and their interference with gene transcription and translation has an effect on TAA, AMP and TME. Epstein-Barr virus (EBV) and Helicobacter pylori (Hp) infection in GC entail a more sophisticated process of ncRNAs, and this review will not cover it.

Micro RNAs (miRNAs) adversely regulate gene expression by interacting with mRNA 3’-UTR targets, resulting in polyadenylation, decreased mRNA stability, and translational inhibition (150). On the other hand, miRNAs can influence transcription by binding to certain genes in a targeted manner (150). Inhibiting PD-L1 protein translation by binding to the 3’-UTR of PD-L1 mRNA, miR-200c enhances the anti-tumor response by decreasing PD-L1 expression (151). MiR-16-5p, miR-152, miR-375 and miR-570 are other micro RNAs that can inhibit PD-L1 expression (54, 60, 152–154). In particular, miR-152 is down-regulated by TGF-β and can improve immunological recognition by targeting the 3’-UTR of HLA-G and PD-L1 mRNA (60, 152). By inactivating JAK2, a common upstream inhibitor of STAT3, miR-375 suppresses the JAK2/STAT3 pathway to down-regulate PD-L1 (153). Also, miR-588, miR-29a-3p, miR-34a and miR-30c increase anti-tumor immune response by promoting CD8+ T cell, M1 macrophage, B cells, GZMB and IFN-γ infiltration (55, 155–157). It has been shown that CAFs infiltration can be suppressed by miR-141-3p, which can directly target STAT4 to inhibit its expression and restrict the Wnt/β-catenin pathway (158). On the contrary, miR-1920 and miR-675-3p could up-regulate the expression of PD-1/PD-L1 (159, 160). There is a drop in CD8+ T cells and NK cells in response to miR-494, miR-1269a and miR-17-5p, but an increase in MDSCs, M2 macrophages, tolerance DCs and Treg infiltration (56, 64, 76). A protein called monocyte chemoattractant protein-1 (ZC3H12A) is encoded by this gene, which has anti-tumor effects because it suppresses chronic inflammation. Mir-425-3p can directly target this gene to boost the inflammatory response and facilitate immune evasion (161).

Sponging with miRNAs to interfere with its function, influencing downstream targets, is also relevant for long noncoding RNAs (lncRNAs) and circular RNAs (circRNAs). For instance, linc00936 works together with miR-425-3p to enhance the body’s natural anti-inflammatory response by increasing ZC3H12A expression (161). Oppositely, lncRNA POU3F3, HOTAIR, MALAT1, H19, MIR100HG and linc00963 favored immunological escape by down-regulating IL-21R, Treg, M2 macrophages and TGF-β and up-regulating the infiltration of mature DCs, CD8+ T cells, M1 macrophages and IFN-γ (57, 77, 162–166). Additionally, PD-L1 expression was boosted by SNHG15 and UCA1 through spongingmiR-141, miR-193a and miR-214, respectively (167, 168). There has been little exploration into the role of circRNAs in GC immune escape, but this could be an exciting new field of study. **Table 2** summarizes this paper’s discussion of the role of ncRNAs as regulatory mechanisms in GC immune evasion.

**Table 2:** Impact of epigenetic regulation on immune evasion in GC.

	Mechanism	Effect	Ref
miR-200c	down-regulating PD-L1 expression	anti-oncogene	(151)
miR-16-5p	down-regulating PD-L1 expression; increasing Teff infiltration	anti-oncogene	(54)
miR-152	down-regulating HLA-G and PD-L1 expression	anti-oncogene	(60, 152)
miR-375	inactivating JAK2/STAT3 pathway to decrease PD-L1 expression	anti-oncogene	(153)
miR-570	down-regulating PD-L1 expression	anti-oncogene	(154)
miR-588	increasing CD8+ T cellsinfiltration by up-regulating CXCL5/9/10	anti-oncogene	(155)
miR-29a-3p	increasing M1 macrophage and B cell infiltration by targeting COL1A2	anti-oncogene	(156)
miR-34a	reducing lactic acid accumulation in T cells; increasing Teff, IFN-γ and GZMB infiltration	anti-oncogene	(157)
miR-30c	promoting M1 macrophage polarization	anti-oncogene	(55)
miR-141-3p	inhibitingSTAT4/Wnt/β-catenin pathway to decrease CAFs	anti-oncogene	(158)
miR-1290	up-regulating PD-1 expression via Ghl2/ZEB1 axis	oncogene	(159)
miR-675-3p	up-regulating PD-L1 expression via CXXC4/MAPK axis	oncogene	(160)
miR-494	increasing MDSCs infiltration by PTEN/PI3K/Akt axis	oncogene	(64)
miR-1269a	inhibiting CXCL9 expression to increase MDSCs, M2 macrophage and decrease CD8+, CD4+ T, NK and B cells	oncogene	(56)
miR-17-5p	inhibiting DCs endocytosis; promoting Treg differentiation;decreasingTNF-α, IL-12 and increasing IL-10 infiltration	oncogene	(76)
miR-425-3p	amplifying inflammation by targeting ZC3H12A	oncogene	(161)
linc-00936	amplifying anti-inflammatory response *via* sponging miR-425-3p	anti-oncogene	(162)
linc-POU3F3	recruiting TGF-β to activate TGF-β/SMAD2/3 pathway; promoting Treg differentiation	oncogene	(163)
linc-00963	inhibiting DCsmaturationvia miR-612/CDC5L axis	oncogene	(77)
lncRNA HOTAIR	up-regulating COL5A1 to decrease the infiltration of CD8+ T cell, M1 macrophage, neutrophils and mature DCs by sponging miR- 1277-5p; up-regulating CXCR4 by sponging miR-126	oncogene	(164, 165)
lncRNA MALAT1	up-regulating IL-21R/JAK2/STAT3 *via* sponging miR-125a	oncogene	(57)
lncRNA H19	attenuating Teff(especially Th1 andCD8+ T cells), NK cell functionand increasing M2 macrophage number by activating LDHA	oncogene	(166)
lncRNA MIR100HG	decreasing Teffand IFN-*γ*viaactivating ERK1/2	oncogene	(167)
lncRNA SNHG15	up-regulating PD-L1 expression *via* sponging miR-141	oncogene	(168)
lncRNA UCA1	up-regulating PD-L1 expression *via* sponging miR-193a, miR-214	oncogene	(150)

## 4 Perspectives

Despite progress in gene sequencing technology and the promise of precision medicine, there are still too many examples when treatment causes more harm than good. The primary goals of modern immunotherapy are Teff function restoration and ICPs inhibition (169). In recent years, however, monotherapy has been found to have drawbacks; For example, RhoA Y42 mutant GC is not a viable candidate for PD-1 blocking monotherapy (119). Furthermore, several signaling pathway components, such as EGFR, HER2 and VEGF, have become effective therapeutic targets because of their crucial involvement in GC (170). Specially, the HER2 inhibitor, Trastuzumab, can reduce the activity of the PI3K/AKT pathway, which is responsible for the uncontrolled growth of tumor cells, and so restore innate antitumor immunity (12, 13, 170). Tumor cell proliferation, invasion, migration, and the development of an immunosuppressive TME can all be stifled by inhibiting these signaling pathways. In addition, ncRNAs are treated in two major ways, either as an alternative therapy or an inhibitory therapy, each of which could be used as a therapeutic target (171). Hence, research into immunotherapies that use molecularly targeted drugs in tandem with conventional ones has great potential in the future.

## 5 Conclusion

Tumor immune evasion, which includes TAA insufficiency, APM abnormalities, TME composition changes, etc., is a significant research field. Tumor cells’ immunogenicity can be changed by even a little change in the antigen, allowing them to evade immune detection. Tumor formation and spread into the TME are aided by the early microenvironment once the tumor escapes immune monitoring. We present a brief overview of the immune evasion pathways associated with GC that can be used as immunotherapy targets. While the immune evasion process may be complicated, it must be deciphered in order to provide targeted care. More research and clinical trials are needed to better understand immune evasion, particularly in relation to the prognosis of GC and the development of new therapeutic options for the many distinct subtypes of the disease.

## Author contributions

JW, TL and TH wrote the draft review, JW was responsible for responding to the reviewer’s questions and revising the paper, MS was involved in literature search and curation, XW was involved in original idea and critical revision of the manuscript. All authors contributed to the article and approved the submitted version.

## Funding

This research was supported by the Department of Science and Technology of Jilin Province (20200708109YY).

## Conflict of interest

The authors declare that the research was conducted in the absence of any commercial or financial relationships that could be construed as a potential conflict of interest.

## Publisher’s note

All claims expressed in this article are solely those of the authors and do not necessarily represent those of their affiliated organizations, or those of the publisher, the editors and the reviewers. Any product that may be evaluated in this article, or claim that may be made by its manufacturer, is not guaranteed or endorsed by the publisher.

## Abbreviations

APCs, antigen-presenting cells; APM, antigen presentation mechanism; CAFs, cancer-associated fibroblasts; CCL, C-C motif chemokine ligand; CCR, C-C motif chemokine receptor; CD, cluster of differentiation; CTLs, cytotoxic T lymphocytes; CTLA-4, cytotoxic T lymphocyte associated protein 4; CXCL, C-X-C motif chemokine ligand; IDO 1, indoleamine 2,3-dioxygenase 1; MDSCs, Myeloid-derived suppressor cells; MHC, major histocompatibility complex; TAA, tumor-associated antigen; TAMs, tumor-associated macrophages; TAP, transporter associated with antigen processing; Teff, effector T cell; TGF-β, transforming growth factor-β; Th cell, helper T cell; TME, tumor microenvironment; TNF-α, tumor necrosis factor-α; Treg, regulatory T cell.
